# Understanding the Intersection of Ageism and Dementia‐Related Stigma During the American Presidential Election: A Thematic Analysis of Social Media Discourse

**DOI:** 10.1002/alz70858_104066

**Published:** 2025-12-25

**Authors:** Juanita‐Dawne R Bacsu, Megan E O'Connell, Ali Akbar Jamali, Megan Funk, Alixe Ménard, Raymond J. Spiteri

**Affiliations:** ^1^ Thompson Rivers University, Kamloops, BC, Canada; ^2^ University of Saskatchewan, Saskatoon, SK, Canada; ^3^ University of Ottawa, Ottawa, ON, Canada

## Abstract

**Background:**

When the United States (U.S.) Presidential Election was announced in 2024, Joe Biden and Donald Trump were two of the oldest candidates in election history. This circumstance contributed to sentiments of ageism and dementia‐related stigma. Although ageism and dementia‐related stigma frequently intersect, there is a paucity of research exploring these interconnected issues. This study used tweets from X (formerly Twitter) to understand the intersection of ageism and dementia‐related stigma on social media during the 2024 U.S. Presidential Election.

**Method:**

We purchased a professional developer account to access the official Application Programming Interface (API) of the social media platform X. We collected relevant tweets from X using the Tweepy application in Python during the U.S. Presidential Election campaign from February 11‐25, 2024. Using filters, 1,254 relevant posts were analyzed using thematic analysis. Actions were taken to support trustworthiness and rigor such as inter‐rater reliability coding and the creation of a thematic map.

**Result:**

Based on our thematic analysis, four main themes were identified: 1) dehumanization of older adults: “ancient fossils are running for office,” 2) dementia‐related ridicule: “demented addled candidate,” 3) old age as an inherent weakness: “they’re both too old; and 4) fear of perceived incompetence: “the fear is palpable.”

**Conclusion:**

Our findings shed light on how social media discourse can threaten the credibility of older political leaders by shifting the focus from campaign policies to dementia‐related stigma and ageism. The persistent questioning of the candidates’ competence, coupled with ageist rhetoric, may undermine the credibility of older political leaders and public trust in the electoral system. Further research is needed to address the impact of ageism and dementia‐related stigma on political leaders’ credibility and the larger electoral system.

